# Preclinical evaluation of the proteasome inhibitor bortezomib in cancer therapy

**DOI:** 10.1186/1475-2867-5-18

**Published:** 2005-06-01

**Authors:** Mario Boccadoro, Gareth Morgan, Jamie Cavenagh

**Affiliations:** 1 Section of Hematology, University of Torino, Torino, Italy; 2 Royal Marsden Hospital, Surrey, UK; 3 St. Bartholomew's Hospital, Department of Haematology, London, UK

## Abstract

Bortezomib is a highly selective, reversible inhibitor of the 26S proteasome that is indicated for single-agent use in the treatment of patients with multiple myeloma who have received at least 2 prior therapies and are progressing on their most recent therapy. Clinical investigations have been completed or are under way to evaluate the safety and efficacy of bortezomib alone or in combination with chemotherapy in multiple myeloma, both at relapse and presentation, as well as in other cancer types. The antiproliferative, proapoptotic, antiangiogenic, and antitumor activities of bortezomib result from proteasome inhibition and depend on the altered degradation of a host of regulatory proteins. Exposure to bortezomib has been shown to stabilize p21, p27, and p53, as well as the proapoptotic Bid and Bax proteins, caveolin-1, and inhibitor κB-α, which prevents activation of nuclear factor κB-induced cell survival pathways. Bortezomib also promoted the activation of the proapoptotic c-Jun-NH_2 _terminal kinase, as well as the endoplasmic reticulum stress response. The anticancer effects of bortezomib as a single agent have been demonstrated in xenograft models of multiple myeloma, adult T-cell leukemia, lung, breast, prostate, pancreatic, head and neck, and colon cancer, and in melanoma. In these preclinical *in vivo *studies, bortezomib treatment resulted in decreased tumor growth, angiogenesis, and metastasis, as well as increased survival and tumor apoptosis. In several *in vitro *and/or *in vivo *cancer models, bortezomib has also been shown to enhance the antitumor properties of several antineoplastic treatments. Importantly, bortezomib was generally well tolerated and did not appear to produce additive toxicities when combined with other therapies in the dosing regimens used in these preclinical *in vivo *investigations. These findings provide a rationale for further clinical trials using bortezomib alone or in combination regimens with chemotherapy, radiation therapy, immunotherapy, or novel agents in patients with hematologic malignancies or solid tumors.

## Introduction

Bortezomib (VELCADE^®^, formerly PS-341) was approved for the treatment of patients with relapsed or refractory multiple myeloma in May 2003 by the US Food and Drug Administration [1] and in April 2004 by the Committee for Proprietary Medicinal Products of the European Union. A number of clinical studies evaluating the activity and safety of bortezomib in multiple myeloma, as well as in other types of cancer, have been conducted [2-10] or are ongoing [11]. Therefore, a review of key preclinical studies that have explored the mechanisms of action and provided the rationale for clinical investigation of this novel agent in multiple myeloma and other cancer types is warranted.

## Mechanism of action

**Figure 1:**
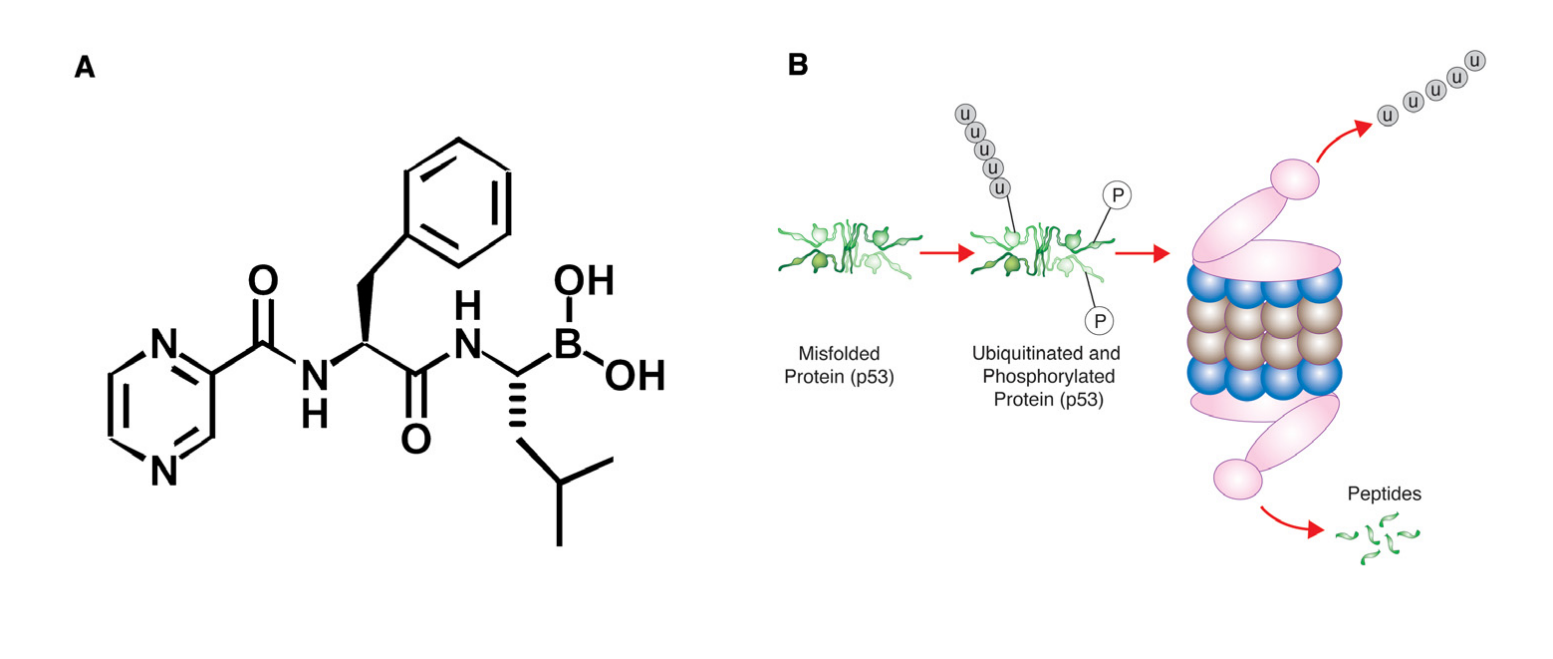
Chemical structure of the proteasome inhibitor bortezomib: pyrazylcarbonyl-Phe-Leu-boronate (A). Schematic illustration of the ubiquitin-proteasome pathway. Misfolded proteins (e.g., p53) are targeted for degradation by the 26S proteasome via phosporylation and ubiquitination (B). Following substrate degradation, the ubiquitin tags and peptides are recycled for future use.

**Figure 2:**
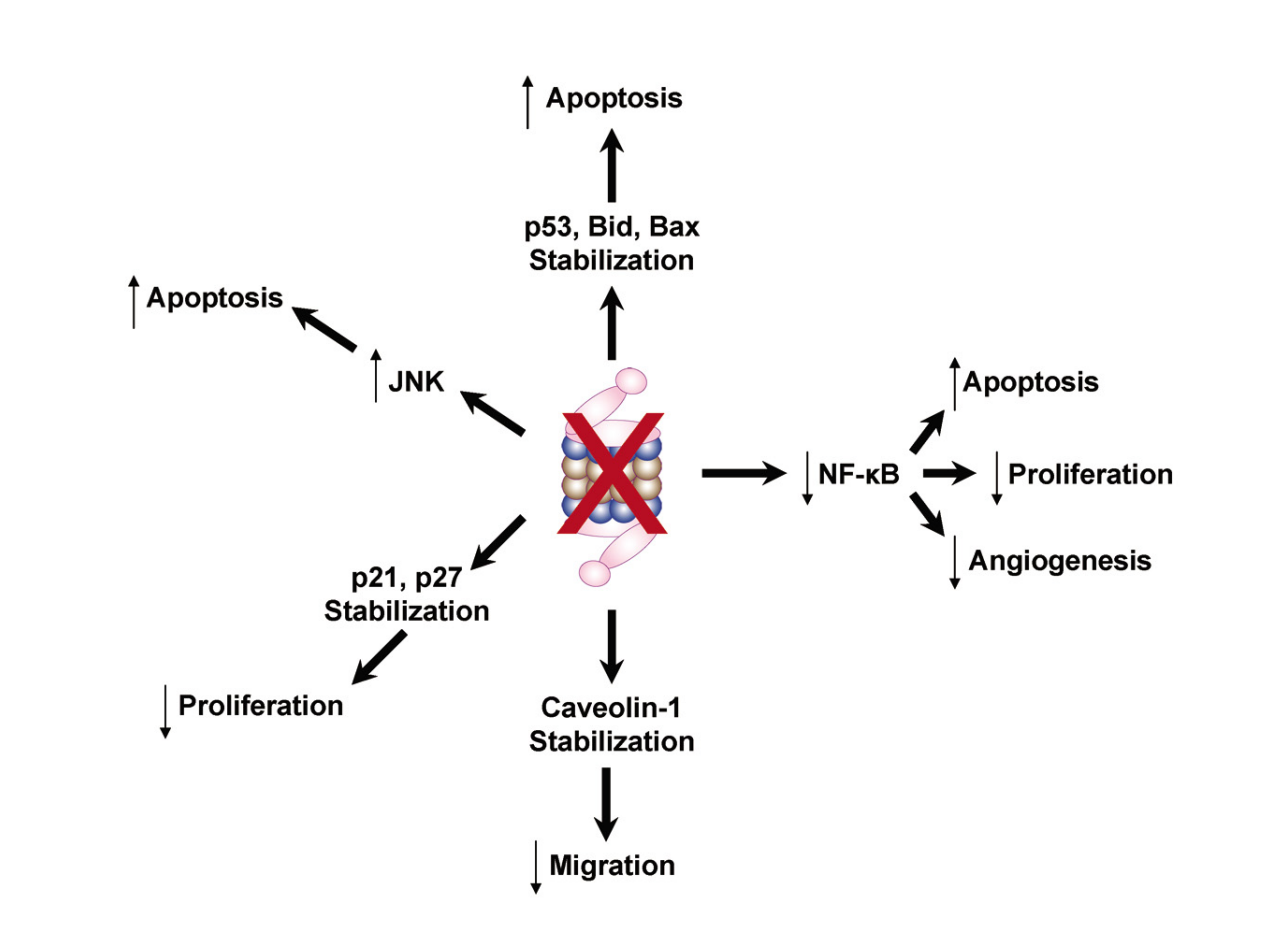
Inhibition of the proteasome by bortezomib results in activation of JNK, stabilization of p53, Bid, Bax, p21, p27, caveolin-1, and IκBα, resulting in inhibition of NF-κB. Alteration of the levels of these cellular proteins leads to inhibition of proliferation, migration, and angiogenesis and promotion of apoptosis of cancer cells.

Bortezomib, a boronic acid dipeptide (Figure 1A) [12], is a highly selective, reversible inhibitor of the 26S proteasome that was first shown to exhibit antitumor properties in a panel of 60 cancer cell lines from the US National Cancer Institute [13]. The proteasome is an enzyme complex that primarily functions in the degradation of misfolded proteins and is essential for the regulation of the cell cycle. Proteasomes are localized in the nucleus and cytosol, where they are largely associated with centrosomes, the cytoskeleton, and the outer endoplasmic reticulum [14]. Damaged intracellular proteins are targeted for elimination by the proteasome through ubiquitination (Figure 1B). Many of the substrates that have been identified are proteins that function in the regulation of transcriptional activation, signal transduction, cell cycle proliferation, and apoptosis (Figure 2, Table 1) [15-29].

**Table 1:** Intracellular targets of bortezomib.

**Protein**	**Function**	**Effect of bortezomib**	**References**
IκBα	Regulates the activity of the transcription factor, NF-κB	Stabilization	Hideshima et al [15], 2001 Russo et al [16], 2001 Sunwoo et al [17], 2001 Hideshima et al [18], 2002 Tan and Waldmann [19], 2002 Ma et al [20], 2003
JNK	Phosphorylates and activates the transcription factor c-Jun	Activation	Hideshima et al [21], 2003 Chauhan et al [22], 2004 Yang et al [23], 2004
p21, p27	CDK inhibitors	Stabilization	Shah et al [24], 2001 Hideshima et al [15], 2001 Yang et al [23], 2004
p53	Transcription factor and Tumor suppressor	Stabilization	Williams and McConkey [25], 2003 Hideshima et al [21], 2003 Yang et al [23], 2004
Bid	Proapoptotic protein	Stabilization	Breitschopf et al [26], 2000
Bax	Proapoptotic protein	Stabilization	Li and Dou [27], 2000
Caveolin-1	Promotes cell migration	Inhibition of activation	Podar et al [28], 2004
gp130	Cytokine signaling receptor	Downregulation	Hideshima et al [29], 2003
DNA-PKcs	DNA repair	Cleavage	Hideshima et al [21], 2003
ATM	DNA repair	Cleavage	Hideshima et al [21], 2003

IκBα = inhibitor κB-α; JNK = c-Jun-NH_2 _terminal kinase; CDK = cyclin-dependent kinase; DNA-PKcs = DNA protein kinase catalytic subunit; ATM = ataxia telangiectasia, mutated.

Several independent investigators have found that bortezomib inhibits activation of the transcription factor nuclear factor κB (NF-κB) [15-20,30]. NF-κB is important for cell survival and is activated in response to cell stress, including that induced by cytotoxic agents, radiation, or DNA damage. NF-κB is overexpressed in several tumors and regulates the expression of genes involved in apoptosis (including Bcl-2 and Bcl-xL), cell cycle progression, inflammation, and angiogenesis (including interleukin [IL]-6, IL-8, and vascular endothelial growth factor [VEGF]) [31-33]. NF-κB is normally bound in the cytosol to inhibitor κB-α (IκBα). Phosphorylation, ubiquitination, and degradation of IκBα are required for NF-κB to translocate to the nucleus and activate the transcription of target genes. Bortezomib blocks the activation of NF-κB by preventing proteasomal degradation of IκBα. Through inhibition of NF-κB, bortezomib not only promotes apoptosis of cancer cells but also sensitizes these cells to chemotherapy [15,20,30], radiation [16], or immunotherapy [19]. However, because specific NF-κB inhibition alone via PS-1145 only partially inhibits proliferation of tumor cells [18], the cytotoxic activity of bortezomib must also depend on altered regulation of other signal transduction pathway targets [18].

The intracellular levels of a number of other proteins that regulate gene transcription, apoptosis, and proliferation are significantly affected by bortezomib (Table 1). c-Jun-NH_2 _terminal kinase (JNK) is a protein that promotes cell death in response to stress and increased levels of misfolded proteins [34]. Bortezomib treatment leads to activation of JNK in multiple myeloma [21,22] and non-small cell lung cancer cells [23]. These studies further showed that specific inhibition of JNK activation, either genetic or pharmacologic, prevented mitochondrial release of cytochrome c and Smac, activation of caspase-8, -9, and -3, and apoptosis.

Proteasome inhibition has also been shown to stabilize the cyclin-dependent kinase inhibitors p21 and p27, the tumor suppressor p53, and the proapoptotic proteins Bid and Bax [15,21,23-27]. The increased levels of activated p21, p27, p53, Bid, and Bax result in inhibition of cell cycle progression and/or promotion of apoptosis in response to bortezomib. Interestingly, sensitivity to proteasome inhibition was partially dependent on the p53 status of breast [35] and lung cancer *in vitro *[36], but bortezomib-induced apoptosis and/or chemosensitization were p53 independent in prostate [13], multiple myeloma [15], and colon cancer cells [30]. Therefore, the degree of variability in the sensitivity to bortezomib with respect to p53 status appears cell-type dependent.

A recently published study found that bortezomib prevented activation of caveolin-1 in multiple myeloma cells [28]. Activation of caveolin-1, a protein that functions in cell motility or migration in a number of tissues, requires phosphorylation. In this report, bortezomib was shown to prevent phosphorylation of caveolin-1 by VEGF, a proangiogenic cytokine and transcriptional target of NF-κB [37]. Bortezomib also inhibited VEGF secretion by the bone marrow. Together, these findings demonstrate important mechanisms by which bortezomib may inhibit migration of cancer cells as well as tumor angiogenesis.

The specific proteins mentioned have all been shown to be at least partially responsible in various models for the antiproliferative, proapoptotic, antiangiogenic, and antitumor effects of bortezomib. However, recent studies have found that bortezomib results in cytotoxic activity through activation of the endoplasmic reticulum stress response [38-41]. The mechanism appears to involve blockade of retrograde transportation and degradation of damaged endoplasmic reticulum proteins by proteasome inhibition [42]. Further studies are necessary to link these new findings with the specific intracellular signals that have been previously implicated in the anticancer activities of bortezomib.

## Bortezomib alone

Bortezomib has shown promising antitumor activity in a number of preclinical cancer murine models *in vivo *(Table 2) [13,17,19,24,30,43-49]. In a xenograft model of multiple myeloma, bortezomib treatment resulted in significant inhibition of tumor growth, an increase in overall survival, and a decrease in tumor angiogenesis [43]. The proteasome inhibitor was well tolerated up to 0.5 mg/kg intravenously (IV) twice weekly for 4 weeks, with dose-limiting toxicities, including weight loss, at 1 mg/kg. Two recent reports evaluating the efficacy of bortezomib in murine xenograft models of adult T-cell leukemia have reached contradictory conclusions. Tan and Waldman found that bortezomib treatment alone, 0.06 mg/kg intraperitoneally (IP), daily for 3 weeks, did not produce significant antitumor effects [19]. However, a second group reported that bortezomib 1.0 mg/kg IP twice weekly for 2 weeks resulted in antitumor activity [44]. Whereas Tan and Waldman reported lethality at a more aggressive dosing regimen of bortezomib 0.1 mg/kg IP twice daily for 2 weeks, slight, temporary weight loss was the only adverse effect described by the second group of investigators [44]. It seems plausible that the discrepancy in activity and toxicity may be due to the differences in the doses and dosing regimens. Based on the finding that proteasome inhibition by bortezomib lasts for up to 72 hours [8], more recent preclinical studies have used twice-weekly, rather than daily or twice-daily dosing, and have shown overall greater activity with less toxicity.

**Table 2:** Activity of bortezomib in tumor models *in vivo*.

**Cancer**	**Activity**	**MTD**	**References**
Multiple myeloma	Decreased tumor growth and angiogenesis; increased survival	0.5 mg/kg IV twice weekly for 4 weeks	LeBlanc et al [43], 2002
Adult T-cell leukemia	Decreased or no effect on tumor growth	1.0 mg/kg IP twice weekly for 2 weeks	Tan and Waldmann [19], 2002 Satou et al [44], 2004
Lung	Tumor growth delay and decreased lung metastases	1.0 mg/kg PO once daily for 18 days	Teicher et al [45], 1999
Breast	Decreased surviving fraction of tumor cells	5.0 mg/kg IP once	Teicher et al [45], 1999
Prostate	Decreased tumor growth; decrease or no effect on angiogenesis	1.0 mg/kg IV weekly for 4 weeks, or q 72 hrs for 15 days	Adams et al [13], 1999 Williams et al [46], 2003
Pancreatic	Decreased or no effect on tumor growth and angiogenesis; increased or no effect on tumor apoptosis	1.0 mg/kg IV biweekly for 2 to 3 weeks, or weekly for 4 weeks or 0.25 mg/kg IP biweekly for 4 weeks	Shah et al [24], 2001 Nawrocki et al [47], 2002 Bold et al [48], 2001
Head and neck	Decreased tumor growth and angiogenesis	1.5 mg/kg IP 3 times per week for 3 weeks	Sunwoo et al [17], 2001
Colon	Decreased tumor growth and increased tumor apoptosis	1.0 mg/kg IV twice weekly	Cusack et al [30], 2001
Melanoma	Decreased tumor growth and angiogenesis; increased tumor apoptosis	1.25 mg/kg SC twice weekly for 5 weeks	Amiri et al [49], 2004

MTD = maximum tolerated dose; IV = intravenous; IP = intraperitoneal; PO = by mouth; SC = subcutaneous.

An evaluation of the effects of bortezomib in murine xenograft models of both lung and breast cancer was also conducted [45]. Treatment with oral bortezomib 1.0 mg/kg daily for 18 days caused tumor growth delays, as well as a decrease in the number of metastases in the Lewis lung cancer model. Furthermore, in a murine model, bortezomib at a single IP dose of up to 5 mg/kg significantly decreased the surviving fraction of breast tumor cells. A decrease in the level of colony-forming-unit granulocyte macrophages was the only toxicity noted in these experiments.

Two groups of investigators have evaluated the efficacy of bortezomib in murine xenograft models of prostate cancer. The first study concluded that bortezomib 1.0 mg/kg IV weekly for 4 weeks reduced tumor growth by 60% [13]. The second study, in which bortezomib 1.0 mg/kg IV every 72 hours for 15 days was administered, produced similar results, with 50% and 80% inhibition in tumor growth in two xenograft models [46]. This report further showed that bortezomib significantly inhibited tumor angiogenesis in one of the models, as measured by a decrease in the number of CD31+ vessels in tumor sections. No toxicities were detected in either of these studies.

In a study of pancreatic cancer murine xenografts, treatment with bortezomib 1.0 mg/kg IV or IP weekly for 4 weeks resulted in a 72% or 84% reduction in tumor growth, as well as an increase in tumor cell apoptosis, with no evidence of toxicity [24]. Another group found that bortezomib 1.0 mg/kg IV biweekly for 2 to 3 weeks significantly inhibited tumor growth and angiogenesis and promoted apoptosis in 1 of 2 pancreatic cancer xenograft murine models [47]. These investigators also reported adverse events, including decreased body weight, diarrhea, and gastrointestinal inflammation at doses above 1.0 mg/kg and lethality at doses above 1.5 mg/kg. Finally, bortezomib produced significant antitumor, proapoptotic, and/or antiangiogenic effects in murine xenograft models of head and neck [17] and colon cancer [30], as well as melanoma [49]. Adverse events, including dehydration, lethargy, weight loss, and death, were noted at doses of 2.0 mg/kg IP 3 times weekly for 3 weeks in one of these studies [17], whereas the other studies did not report any toxicities at lower doses (Table 2).

These preclinical investigations have collectively demonstrated the antitumor activity of bortezomib as a single agent at tolerable levels in a variety of murine cancer models. However, because the standard approach to antineoplastic therapy generally involves the administration of more than one agent or modality in an effort to prevent the development of chemoresistance and increase tumor cell kill, the effects of bortezomib in combination with chemotherapy, radiation, immunotherapy, or novel agents have been investigated *in vitro *and/or *in vivo*.

## Combination therapy

A number of preclinical studies have evaluated the activity of bortezomib in combination with other therapies (Table 3) [15,16,19,20,24,30,39,40,45,48-61]. The finding that several cytotoxic agents as well as radiation [62,63] activate NF-κB is a major rationale for combining proteasome inhibitors with these therapies in the treatment of cancer. Activated NF-κB is free to translocate to the nucleus and induce the expression of proinflammatory and antiapoptotic genes, such as Bcl-2 and Bcl-xL, which promote tumor cell survival [31-33]. Furthermore, inhibition of NF-κB has been implicated as an important mechanism by which bortezomib sensitizes tumor cells to various drugs or radiation [15,16,20,24,30,48-50,55,57,59,60].

**Table 3:** Evaluation of bortezomib in combination with other therapies.

**Type of therapy**	**Drug or agent**	**Cancer**	**References**
Chemotherapy	5-Fluorouracil, cisplatin, paclitaxel, doxorubicin, cyclophosphamide	Breast, lung	Teicher et al [45], 1999
	Melphalan, doxorubicin, dexamethasone	Multiple myeloma	Hideshima et al [15], 2001 Mitsiades et al [50], 2003 Ma et al [20], 2003
	Topoisomerase inhibitor: irinotecan	Colon Pancreatic	Cusack et al [30], 2001 Shah et al [24], 2001
	Gemcitabine	Pancreatic Bladder	Bold et al [48], 2001 Kamat et al [51], 2004
	Pegylated liposomal doxorubicin	Breast	Small et al [52], 2004
	Docetaxel	Pancreatic	Nawrocki et al [53], 2004
	Temozolomide	Melanoma	Amiri et al [49], 2004
Radiation therapy		Breast Colon Prostate	Teicher et al [45], 1999 Russo et al [16], 2001 Pervan et al [54], 2001
Immunotherapy	Daclizumab	Adult T-cell leukemia	Tan and Waldmann [19], 2002
Novel agents	TRAIL/Apo2L	Multiple myeloma, myeloid leukemia, renal	Mitsiades et al [55], 2001 Sayers et al [56], 2003
	HSP90 inhibitor: 17-AAG	Breast	Mimnaugh et al [39], 2004
	HDAC inhibitors: SAHA, sodium butyrate	CML, multiple myeloma, lung	Denlinger et al [40], 2004 Yu et al [57], 2003 Mitsiades et al [58], 2004 Denlinger et al [59], 2004 Pei et al [60], 2004
Transplantation	Allogeneic BMT	Leukemia	Sun et al [61], 2004

HSP = heat shock protein; HDAC = histone deacetylase; CML = chronic myelogenous leukemia; BMT = bone marrow transplantation.

## Bortezomib with chemotherapy

In an investigation of lung cancer, bortezomib in combination with chemotherapeutic agents, including 5-fluorouracil, cisplatin, paclitaxel, or doxorubicin, produced additive antitumor and antimetastatic effects [45]. In this same study, bortezomib also increased tumor-cell killing by cyclophosphamide and cisplatin, as well as tumor-cell killing by radiation, in an *in vitro*-*in vivo *model of breast cancer. Although toxicity is difficult to assess in preclinical models, no added toxicities were observed when bortezomib was added to the other therapies, and bortezomib dose modifications were not required.

Several studies evaluating the effects of bortezomib in combination with other therapies have been conducted in multiple myeloma. These *in vitro *experiments collectively confirmed that bortezomib enhanced the antiproliferative and proapoptotic effects of conventional antimyeloma agents, including melphalan, doxorubicin, and dexamethasone [15,20,50]. These investigators also reported that multiple myeloma cell lines that were previously resistant to melphalan, doxorubicin, dexamethasone, or mitoxantrone were sensitized up to 1,000,000-fold by prior exposure to subtoxic concentrations of bortezomib. Finally, the researchers showed that bortezomib was not only directly cytotoxic to the multiple myeloma cells but that it also altered the microenvironment through inhibition of IL-6 to prevent the growth of tumor cells in proximity to the bone marrow [15].

Two groups of researchers examined the effects of bortezomib in combination with the topoisomerase inhibitor irinotecan in murine xenograft models of colon [30] and pancreatic [24] cancer. Both these studies concluded that combined inhibition of the proteasome and topoisomerase resulted in enhanced antiproliferative and proapoptotic effects. The combination of bortezomib and irinotecan therapy further resulted in a 94% [30] or 89% [24] decrease in tumor size compared with controls. These tumors were also significantly smaller than those of the mice receiving either bortezomib (26% or 65% decrease in tumor size relative to controls) or irinotecan (48% or 43% decrease in tumor size relative to controls) as single agents.

Xenograft models of pancreatic cancer were also used to evaluate the activity of bortezomib in combination with other standard chemotherapies [48,53]. Inhibition of the proteasome increased the sensitivity of tumors to both gemcitabine [48] and docetaxel [53], because bortezomib in combination with these agents resulted in significant enhancement of antiproliferative, proapoptotic, antitumor, and/or antiangiogenic activities.

The combination of bortezomib with temozolomide was studied in models of malignant melanoma [49]. Bortezomib enhanced the antiproliferative and cytotoxic effects of temozolomide in melanoma cells *in vitro*, and this combined therapy produced complete remission of tumors lasting more than 200 days in murine xenografts *in vivo*. In contrast, tumors eventually progressed in mice receiving either drug alone. Although specific toxicity evaluations were not performed, no toxicities were observed in any of these investigations.

## Bortezomib with radiation, immunotherapy, or novel agents

A number of studies have reported on the radiosensitizing properties of bortezomib. In addition to the previously mentioned study in breast cancer [45], other laboratories have demonstrated that bortezomib sensitizes colon [16] and prostate [54] cancers to radiation-induced cytotoxicity. In both clonogenic cell survival assays and murine xenograft tumor models, pretreatment with bortezomib enhanced the anticancer effects of irradiation with no observed toxicity.

Another murine xenograft model was used to investigate the effects of bortezomib in combination with daclizumab, a humanized anti-IL-2Rα antibody in adult T-cell leukemia [19]. Although either agent alone resulted in partial or no responses, bortezomib plus daclizumab resulted in prolongation of the survival of tumor-bearing mice. The only adverse effect of the combined therapy was a slight temporary weight loss during the treatment regimen.

Bortezomib has also demonstrated enhanced *in vitro *and/or *in vivo *anticancer effects when combined with novel agents such as TRAIL/Apo2L, a cell death-inducing ligand [55,56], 17-N-allylamino-17-demethoxygeldanamycin (17-AAG), an inhibitor of heat shock protein 90 [39], and suberoylanilide hydroxamic acid (SAHA) or sodium butyrate, both histone deactylase inhibitors [40,57,59,60]. Pretreatment with bortezomib sensitized multiple myeloma, myeloid leukemia, and renal cancer cells but not normal B lymphocytes to TRAIL/Apo2L-induced apoptosis [55,56]. In an *in vivo *experiment, bone marrow and renal cancer cell mixtures, with or without bortezomib and/or TRAIL/Apo2L, were transplanted into the bone marrow of mice. Whereas all the mice receiving cells treated with TRAIL/Apo2L died of leukemia within 35 days, 50% of those receiving cells treated with bortezomib and 90% of those receiving cells treated with both TRAIL/Apo2L and bortezomib survived more than 100 days [56]. A mild transient thrombocytopenia was the only toxicity observed in this study. Finally, while the combination of bortezomib and 17-AAG, SAHA, or sodium butyrate resulted in synergistic antiproliferative and proapoptotic effects *in vitro *[39,40,57-60], these combinations have yet to be evaluated in tumor xenograft models *in vivo*.

## Sequence of administration

One of the areas of controversy has been the appropriate timing of therapy with bortezomib in relation to other antineoplastic agents. A brief discussion of some of the conflicting results is warranted as these preclinical studies may ultimately provide the rationale for clinical trials. In vitro experiments conducted by Mitsiades and colleagues revealed that optimal antimyeloma activity was achieved when bortezomib was administered 24 hours after doxorubicin [50]. The sequence of chemotherapy (gemcitabine) followed by bortezomib was also found to be the most effective in a model of pancreatic cancer [64]. In contrast, Pei et al showed that bortezomib followed by a second antineoplastic agent (SAHA) yielded the highest level of apoptosis in a model of myeloma [60], and Mimnaugh and colleagues found that maximal antiproliferative effects were observed upon simultaneous administration of bortezomib and a heat shock protein 90 inhibitor in a model of breast cancer [39]. It is important to note that these studies utilized bortezomib in combination with different antineoplastic agents in unique cancer models. Further studies may elucidate the reasons for the inconsistent findings.

## Conclusion

The proteasome inhibitor bortezomib exhibits antiproliferative, proapoptotic, antiangiogenic, and antitumor activities in several cancer models. The mechanism of action of bortezomib involves stabilization of NF-κB, p21, p27, p53, Bid, and Bax, inhibition of caveolin-1 activation, and activation of JNK as well as the endoplasmic reticulum stress response. These preclinical evaluations have found that bortezomib is well tolerated at doses that demonstrated antitumor activity in xenograft models of multiple myeloma, adult T-cell leukemia, cancer of the lung, breast, prostate, pancreas, head and neck, and colon, as well as melanoma. Bortezomib also enhances the anticancer effects of chemotherapy, radiation therapy, immunotherapy, or novel agents, without added toxicities requiring dose modifications. The studies provide a rationale for clinical trials of bortezomib alone or in combination with other therapies in patients with solid tumors or hematologic malignancies.

## List of abbreviations

NF-κB = nuclear factor-κB; IκBα = inhibitor κB-α; VEGF = vascular endothelial growth factor; ER = endoplasmic reticulum.

## Competing interests

M.B. has received consulting and lecture fees from Millennium Pharmaceuticals, Inc. G.M. declares that he has no competing interests; he is supported by the Leukaemia Research Fund and the International Myeloma Foundation. J.C. has received advisory board and speakers' bureau fees from Millennium Pharmaceuticals, Inc. and Ortho Biotech.

## Authors' contributions

M.B. reviewed the literature and drafted the manuscript. G.M. and J.C. reviewed and revised the manuscript. All authors read and approved the final version.
